# Do cognitive interventions for preschoolers improve executive functions and reduce ADHD and externalizing symptoms? A meta-analysis of randomized controlled trials

**DOI:** 10.1007/s00787-020-01627-z

**Published:** 2020-09-05

**Authors:** Ursula Pauli-Pott, Christopher Mann, Katja Becker

**Affiliations:** 1 Department of Child and Adolescent Psychiatry, Psychosomatics and Psychotherapy, Philipps-University of Marburg, Hans Sachs Str. 6, 35039 Marburg, Germany; 2 Center for Mind, Brain and Behavior (CMBB), University of Marburg and Justus Liebig University Giessen, Hans-Meerwein-Straße 6, 35032 Marburg, Germany

**Keywords:** Executive functions, Preschool children, Attention-deficit hyperactivity disorder, Oppositional defiant disorder, Intervention, Meta-analysis

## Abstract

Many interventions targeting executive function (EF) development in the preschool period, where malleability might be particularly high, have been created and evaluated. We conducted a meta-analysis of randomized controlled trials (RCTs) on the effects of these interventions on (a) EFs in preschool children from the general population as well as preschool children with (symptoms of) attention-deficit hyperactivity disorder (ADHD) and oppositional defiant disorder (ODD), and (b) ADHD and ODD symptoms in preschool children with ADHD/ODD (symptoms). Literature search yielded 35 RCTs. Risk of bias of the individual studies was assessed. A random-effects model was used. Moderator effects were tested using mixed model analyses. The overall effects on EFs were: *d* = 0.46 (95% CI 0.30–0.61) for working memory (WM), *d* = 0.30 (95% CI 0.21–0.38) for inhibitory control (IC), *d* = 0.33 (95% CI − 0.04 to 0.71) for reward-related IC, and *d* = 0.47 (95% CI 0.28–0.66) for flexibility. In children with ADHD/ODD, mean effects were *d* = 0.64 (95% CI 0.31–0.96) for WM and *d* = 0.46 (95% CI 0.07–0.84) for IC. Studies on reward-related IC and FL were lacking. Effects on ODD and ADHD symptoms were *d* = 0.40 (95% CI − 0.23 to 1.03) and *d* = 0.28 (95% CI − 0.08 to 0.64), respectively. Interventions targeting multiple EFs and using interpersonal cognitive scaffolding approaches showed large and statistically significant effects on ADHD and ODD symptoms. In preschool children of the general population and in those with ADHD/ODD (symptoms), interventions led to an improvement of EF performance. In children with ADHD and ODD, cognitive scaffolding interventions were most effective in terms of reducing ADHD and ODD symptoms. However, more well-controlled studies need to be conducted before any firm conclusions can be drawn.

## Introduction

From the preschool years onwards, children with attention-deficit hyperactivity disorder (ADHD), oppositional defiant disorder (ODD), and conduct disorder (CD) have consistently been found to show deficits in executive functions (EFs). Specifically, studies demonstrated reduced inhibitory control (IC) capacity in the “cool”, non-reward-related context and in the “hot”, reward-related context, a low working memory capacity, and low set-shifting ability [1, 2]. These findings converge with structural and functional brain imaging results, which point to deviations of networks known to mediate cool and hot EFs in children with ADHD [3], and in children with ODD/CD [4]. Accordingly, deviations in EFs have been assumed to be causally involved in the pathogenesis of ADHD and ODD/CD [5–7]. Regarding ADHD, for example, multiple causal pathway models assume that sets of gene–gene and gene–environment interactions cause different deviations of neural networks, which lead to deviations in core EFs and in turn to the complex behavioral phenotypes of the disease. The model suggests developmental sequences, in which these neural and cognitive deviations emerge before the complex behavioral symptoms of ADHD. As such, deviations in EFs have been regarded as basic deficits and as intermediating phenotypes of ADHD and ODD, which precede the secondarily developing symptoms of the disorders [5–7].

EFs are regarded as complex cognitive processes that serve purposes of behavioral regulation and goal-directed action [8]. Theoretical concepts have converged in specifying a set of three correlated, but separable, core components [9, 10], i.e. “inhibitory control” (IC) (comprising response inhibition and interference control), “working memory” (WM), and “flexibility” (FL) [10]. Response inhibition (as a component of IC) refers to the ability to deliberately suppress a triggered, prepotent behavior, and to sustain behavior toward a goal, i.e. resisting temptations and not acting impulsively. The interference control component of IC denotes the suppression of competing information and is related to selective attention. Besides these two IC components, “hot” response inhibition, or “delay of gratification” (DoG), has been regarded as a further dissociable aspect of IC [8, 10, 11]. DoG refers to the capacity to suppress a behavioral approach in a reward-related context, i.e. to wait for a reward. Working memory (WM) describes the ability to keep information in mind while mentally manipulating or working with it [10]. Cognitive flexibility (FL) is defined as switching between tasks or looking at a problem from different perspectives [8].

EFs involve the so-called executive control network, i.e. a set of frontal and parietal brain structures including regions of the prefrontal cortex (PFC), the insula, parts of the supplementary motor area and the anterior cingulate, the intraparietal sulcus, and other areas [12, 13]. In preschool ages, this network is assumed to undergo major normative developmental changes [13]. Accordingly, the interrelated core EF components of WM, IC, and FL show major normative developmental improvements in the preschool years. These changes in EFs are influenced by genetic/maturational and environmental conditions [12, 14] and predict diverse positive social adjustment outcomes as well as early emerging ADHD and ODD symptoms [2, 15]. Against this background, efforts have been undertaken to create and evaluate techniques suitable for improving EFs in preschool ages, where malleability of EFs and underlying brain circuitry were assumed to be particularly high [16]. It has been suggested that an early training of EFs can prevent the development of externalizing and ADHD symptoms in children in the general population, and reduce externalizing and ADHD symptoms in preschool children who already show elevated ADHD and/or ODD symptoms [16].

In recent years, many intervention programs targeting EFs have been developed and evaluated. Several years ago, Diamond and Lee [17] reviewed the available studies. The authors distinguished four different types of interventions for (preschool) children: (1) computerized training, such as “Cogmed Training” on WM, (2) mindfulness practices promoting sensory awareness and attention regulation, (3) programs emphasizing cognitive scaffolding such as the “Tools of the Mind” program, and (4) programs focusing on social skills and emotion regulation with a minor component aiming at the modification of core EFs. Diamond and Lee [17] found that regardless of the specific principle of the intervention, children with low EFs, e.g. children from low income, socially disadvantaged families, benefitted the most [17, 18]. However, the generalization of effects from a single EF targeted by the training program to other EFs and other basic cognitive abilities was limited [10, 17].

Meta-analyses of the interventions for preschool children have not yet been performed. However, several meta-analyses on WM training (targeting the short-term memory component of WM) have been conducted. Melby-Lervåg and Hulme [19] analyzed 23 studies on WM training in clinical and non-clinical samples of children, adolescents, and adults, and revealed a large overall mean effect size on verbal WM (*d* = 0.79, *p* < 0.001) and a medium effect on visuospatial WM (*d* = 0.52, *p* < 0.001). In children younger than 10 years, effect sizes for verbal and visuospatial WM were *d* = 1.41 (*p* < 0.01) and *d* = 0.46 (*p* < 0.01), respectively. Transfer effects to the Stroop measure (interference control) and to other cognitive abilities were small. Two further meta-analyses analyzed training effects on EFs in children with ADHD and high ADHD symptoms. Rapport et al. [20] included 25 studies on WM training, training of mixed EFs, and attention training in children with ADHD. On tasks similar to the training tasks, the overall effect size of WM training (eight studies) was medium (*d* = 0.63, *p* < 0.05), and the training of mixed EFs (three studies) showed no noteworthy effect (*d* = 0.06). Far transfer effects on objective outcomes (e.g. cognitive tests) were modest. Wass et al. [21] analyzed the transfer effects of cognitive training and found larger transfer effects in younger ages.

Three recent meta-analyses studied the effects of cognitive training on ADHD symptoms in ADHD patients. Sonuga-Barke et al. [22] included six randomized controlled trials on computerized attention and WM training of children and adolescents with an ADHD diagnosis. The overall weighted mean effect size of these studies on ADHD symptom ratings was *d* = 0.64 (*p* < 0.05). To control for the influences of expectancy effects of patients, parents, and researchers (i.e. more favorable ratings of responses to treatment by those who were invested in the therapy), mean effect sizes were estimated exclusively for studies with (probably) blinded assessments of ADHD symptom outcomes (i.e. symptoms rated by an assessor unaware of participants’ allocation to treatment vs. control group). The weighted mean effect size of the five respective studies was *d* = 0.24 and did not reach statistical significance. The meta-analysis by Rapport et al. [20] analyzed far transfer effects to subjective ratings of child behaviors. Weighted mean effect sizes on unblinded and blinded behavior ratings were *d* = 0.48 (*p* < 0.05) and *d* = 0.12 (not significant), respectively. Comparably, Cortese et al. [23] found a small effect (*d* = 0.37, *p* < 0.05) on ADHD symptoms in children with ADHD and a decrease to *d* = 0.20 (*p* < 0.05) when exclusively blinded studies were considered. These authors suggested a possible advantage of programs targeting multiple EF deficits.

Taken together, there is broad evidence regarding the significance of EFs in social adjustment development and psychopathology. In children with ADHD and externalizing symptoms, poor EF development has frequently been found, and has been assumed to be involved in the development of ADHD and ODD symptoms. Cognitive training of EFs in the preschool period was expected to be particularly effective, including the reduction of ADHD and externalizing symptoms. In recent years, specific intervention programs tailored to preschool children have been developed and evaluated (see description/definition of interventions below). In many cases, these programs focus on multiple core EFs and contain elements which target the facilitation of transfer. However, a meta-analysis summarizing the current results of this research has not yet been conducted.

In the present meta-analysis, we, therefore, examined the following hypotheses: (1) cognitive interventions targeted at EFs (as defined by Diamond and Lee [17]) in preschool children increase the core EFs of WM, IC, reward-related IC, and FL in preschool children from the general population and in preschool children showing ADHD and/or ODD (diagnosis or high symptoms). (2) These interventions reduce ADHD and ODD symptoms in preschoolers with diagnoses or high symptoms of the disorders. Finally, we explore differences in the effect sizes of the four types of intervention approaches distinguished by Diamond and Lee [17].

## Methods

### Identification of studies

To be included in the meta-analysis, a study had to fulfill the following criteria: (1) the study tested the effectiveness of an intervention which was developed to improve EFs in preschool and kindergarten children using a mainly cognitive approach (i.e. one of the four, above-mentioned types of interventions distinguished by Diamond and Lee [17]). (2) The mean age of the sample lay between 3;0 and 6;11 years at baseline. (3) The sample was drawn from the general population of children or children with ADHD, ODD, or externalizing disorders (diagnosis or high symptoms, i.e. questionnaire scores at or above a clinical cut-off [23]). We excluded studies on children with intellectual disability, sensory disabilities, or specific neurological diseases such as epilepsy. In these populations, specific intervention strategies might be necessary and effective, and specific mechanisms might mediate the effects of interventions. (4) At least one of the core EFs or an ADHD, ODD, or externalizing score was used as an outcome variable. (5) A randomized controlled trial (RCT) was conducted. (6) Statistics which allow for the calculation of the effect sizes were reported or provided by authors on request. (7) The study was published in a peer-reviewed journal in English.

The meta-analysis follows the suggestions of the PRISMA statement [24]. A literature search was conducted in the electronic databases Science Citation Index, Social Science Citation Index, PsycINFO, and MEDLINE for the period between the start of the respective database and April, 2018. In two steps, we (a) searched for keywords for intervention (e.g. intervention, prevention, training, treatment, program*, health promotion) combined with keywords for executive functions (e.g. executiv* function*, self regulat*, emotion* regulation, working memory, inhibitor* control*, delay of gratification, delay aversion), and (b) searched for keywords for intervention (see above) combined with keywords for externalizing symptoms (e.g. attention deficit*, hyperactiv*, ADHD, oppositional*, aggress*, external*, expansive). Searches were restricted to preschool age (preschool*, kindergarten, pre-kindergarten, early childhood) and English articles.

### Data collection and coding procedure

Besides information necessary for effect size calculations (see below), the variables listed below (moderator variables section) were coded. For this purpose, a standardized coding sheet was developed. Coding was carried out by two psychologists (first and second author). 20% (*k* = 8) of the studies were coded independently by the two coders to assess inter-coder agreement. Kappa coefficients for the categorical variables ranged between 0.74 and 1.0, and Kendall’s Tau-*b* correlations for the continuous variables ranged between 0.79 and 1.0, indicating excellent inter-coder reliability. Statistics necessary for effect size calculation were extracted by the second author (C.M.) and independently checked by the first author (U.P.-P.) in all cases. Divergence was solved by renewed inspection and discussion where necessary.

As outcome measures, we used the pre- to post-intervention change in core EFs (i.e. WM, cool IC, hot IC, FL), ADHD, and ODD/externalizing symptoms measured at the first post-intervention assessment. In most instances (32 of 35 studies), a pretest–posttest control group design was used. To take advantage of the strengths of this design, the difference between the standardized mean change for the treatment and control group was used as primary effect size (see statistics section for further details).

Several studies used more than one single task to assess one EF or symptom domain to increase the reliability of measurement. In some of the studies, composite scores had already been computed and listed in the article. Otherwise, we calculated the weighted mean effect size of the multiple tests per outcome domain. In cases where multiple scores were reported for a single test (e.g. error and accuracy scores), we used the score which most precisely reflected the outcome domain. If more than one independent study was reported in one article, all eligible studies were coded separately (see Table 1).

**Table 1:** Description of included studies

Study	Outcome measures				Intervention				
	ADHD/ODD sample: *X* = yes–no	Number of cases in TG/CG	Mean age of sample in months	Percentage of boys	WM; IC (c = cool, h = hot); FL; O = oppositional/aggr. symptoms (rated by: i = investigator, p = parent, t = teacher); A = ADHD symptoms (rated by: i = investigator, p = parent, t = teacher)	Intervention named by authors (category of intervention approach: 1 = direct training; 2 = cognitive scaffolding; 3 = attention-directing; 4 = minor EF component)	Duration in weeks (intensity in minutes per week)	Setting: G = group; S = single child	Delivery: Te = teacher; Tr = trainer/therapist
Diamond [30]	–	85/62	62	49	ICc: Flanker	Tools of the Mind (2)	52 (UC)	G	Te
Ford [31]	–	30/30	37	53	ICc: Composite score on IC tasks; WM: forward-digit task	Let’s play in Tandem (2)	52 (UC)	S	Te
Bergman Nutley [32]	–	24/25	51	61	WM: grid task, odd one out of AWMA, word span task	WM training (1)	5 (75)	S	Tr
Tominey and McClelland [33]	–	28/37	55	40	ICc: HTKS	Playgrounds intervention (1)	8 (60)	G	Tr
Röthlisberger et al. [34]a	–	33/38	61	54	ICc: Simple-Flanker; WM: Compl.-Span Task; FL: Mixed-Flanker	Intervention program (1)	6 (150)	G	Te
Röthlisberger et al. [34]b	–	30/34	73	63	ICc, WM, FL: see, Röthlisberger et al. [34]a	Intervention program (1)	6 (150)	G	Te
Blair and Raver [18]	–	416/282	NR	NR	ICc: Flanker, hearts–flowers task; WM: backward digit-span task; FL: DCCS	Tools of the mind (2)	39 (UC)	G	Te
van Dongen [35]	X	25/22	79	77	WM: digit-span Task; Ai, t: ADHD Rating Scale IV	WM training (1)	5 (75)	S	Tr
Kroesbergen [36]	–	30/21	70	61	WM: odd one out, word-span backward of AWMA	WM training (1)	4 (60)	G	Tr
Pears [37]	–	25/14	NR	56	ICc: HTKS	Kids-in-transition program (4)	8 (360)	G	Te
Blakey and Carroll [38]	–	26/28	53	50	ICc: Peg-tapping task; WM: backward word-span task	Training of WM and IC (1)	4 (20)	S	Tr
Dias [39]	–	31/37	72	43	ICc: cancellation test, Stroop task, go/no-go, Simon task; FL: trail making	PIAFEx (2)	12 (225)	G	Te
Flook [40]	–	24/32	56	49	ICc: Flanker; ICh: DoG task; FL: DCCS	Mindfulness-based curriculum (4)	12 (60)	G	Tr
Liu [41]	–	16/15	59	53	ICc: Adapted Day–night stroop Task	IC Training (1)	3 (60)	G	Tr
Re et al. [42]a	X	13/13	63	65	ICc: walk–no walk test; WM: dual request selective task; At: Early ADHD scale	Concentration and self-control (1)	6 (120)	G	Te
Re et al. [42]b	–	6/6	65	42	ICc, WM: see, Re et al. [42]a	Concentration and self-control (1)	9 (120)	G	Te
Schmitt [43]	–	126/150	52	49	ICc: HTKS; FL: card sorting task	Playgrounds Intervention (1)	8 (60)	S	Tr
Tamm and Nakonezny [44]	X	10/9	60	74	ICc, WM, and FL: subscales of BRIEF-P; Ai: SNAP-IV	EF Intervention (2)	12 (60)	G	Tr
Traverso [45]	–	32/43	68	47	ICc: Go/No-Go, Flanker; ICh: delay task, gift wrap task; WM: backward word span, Mr. Cucumber, keep track; FL: dots task	EF Intervention (2)	4 (90)	G	Tr
Volckaert and Noël [46]	–	24/23	60	30	ICc: factor score on traffic lights, Cat–dog–fish, Monster Stroop, HTKS; WM: factor score on Categospan, word-span task, block-tapping task	Inhibition training (2)	8 (90)	G	Tr
Fishbein [47]	–	57/57	NR	NR	ICc: Peg tapping, whack-a-mole; ICh: DoG task	PATHS (4)	24 (UC)	G	Te
Graziano and Hart [48]	X	15/15	62	76	ICc: HTKS task; WM: AWMA; Ot: Behavior Assessment System for Children-2	STP-PreK-advanced (4)	8 (375)	G	Tr
Murray et al. [49]	–	59/41	70	58	ICc: Day–night; ICh: Delay task	Attention training technique (3)	1 (48)	G	Te
Poehlmann [50]	–	12/12	52	46	ICc: HTKS task, Go/No-Go	Mindfulness-based curriculum (4)	12 (60)	G	Tr
Thibodeau [51]	–	39/32	52	46	ICc: Day–night; WM: forward digit-span task; FL: card-sorting task	Fantastical pretend play (2)	5 (75)	G	Tr
Dias and Seabra [52]	–	28/30	72	42	FL: trail-making test	PIAFEx (2)	16 (225)	G	TE
Gade et al. [53]a	–	10/10	62	55	WM: word-span, matrix, object-span task	WM training (1)	2 (75)	S	Tr
Gade et al. [53]b	–	16/15	62	52	WM: word-span, matrix, backward color span	WM training (1)	2 (75)	S	Tr
Gade et al. [53]c	–	10/10	72	50	WM: see, Gade et al. [53]b	WM training (1)	2 (75)	S	Tr
Gade et al. [53]d	–	10/10	61	50	WM: see, Gade et al. [53]b	WM training (1)	2 (60)	S	Tr
Houssa [54]	X	16//16	52	54	ICc, WM: subscales of Childhood EF Inventory; Op: CBCL scale	Inhibition training (2)	8 (90)	G	Tr
Howard et al. [55]a	–	19/21	53	48	ICc: Go/No-Go; WM: Mr. Ant; FL: Card Sorting Task	EF intervention (1)	7 (15)	S	Te
Howard et al. [55]b	–	19/15	51	38	ICc, WM, FL: see, Howard et al. [55]a	EF intervention (1)	9 (15)	S	Te
Joekar [56]	X	14/13	69	100	Ap,t: Child Symptom Inventory-4	Pay attention program (1)	11 (45)	S	Tr
Upshur [57]	–	252/240	53	50	ICc: HTKS; WM: backward digit-span task	Second-step early learning (4)	24 (35)	G	Te

*ANT* attention network task, *AWMA* automated working memory assessment, *CBCL* child behavior checklist, *CG* control group, *DCCS* dimensional change card sorting task, *DoG* delay of gratification, *EF* executive functions, *FL* cognitive flexibility, *HTKS* head–toes–knees–shoulders task, *IC* inhibitory control, *NR* not reported, *PATHS* promoting alternative thinking strategies, *PIAFEx* Intervention Program for Self-Regulation and EFs, *SDQ* strengths and difficulties questionnaire, *SNAP-IV* Swanson, Nolan, and Pelham DSM-IV ADHD Rating Scale, *STP-PreK-advanced* summer treatment program for pre-kindergarteners, *TG* treatment group, *WM* working memory, *UC* unclear

#### Hypothesized moderator variables

Regarding the intervention effects on EFs, a distinction was made between samples of children with ADHD, ODD, or externalizing symptoms vs. samples of children from the general population. As mentioned above, we further distinguished four different approaches to EF modification in preschoolers according to Diamond and Lee [17]: (1) direct (computerized) training of a core executive function. In these programs, mostly one EF is focused on and practiced via increasingly challenging tasks. (2) Approaches relying on interpersonal cognitive scaffolding. These preschool programs usually target multiple EFs, may focus on the acquisition of internal language, and establish symbols and visual reminders to facilitate generalization and transfer to everyday life. Some of these interventions explicitly were based on Vygotsky’s theses. (3) Interventions conveying attention-directing strategies such as mindfulness practices, promoting sensory awareness, and attention regulation. (4) Programs with a minor or ancillary component aiming at core EFs. These programs might be classroom curricula focusing primarily on pre-academic or social skills and emotion regulation. By exploring differences between the intervention approaches, it may be possible to obtain some clues regarding specific effects of the approaches in the EF and ADHD/ODD symptom domains.

#### Further potential moderator variables

In cases of significant heterogeneity of effect sizes, we analyzed potential influences of sample and intervention characteristics. We considered the following sample characteristics: (a) mean age of the sample and (b) percentage of boys. The following intervention characteristics were considered: (a) duration of the intervention in weeks, (b) the intensity of the intervention in minutes per week, (c) the setting (group vs. single child), and (d) the delivery of the intervention by teacher (classroom curriculum) vs. trainer/therapist.

### Risk of bias

Regarding risk of bias, the meta-analysis follows the suggestions of the Cochrane Collaboration’s tool [25] as well as the definitions used by Sonuga-Barke et al. [22] and Cortese et al. [23]. Expectancy effects and blinding were regarded as most important [22, 23]. The following variables were coded for each outcome (see, Table 2): (a) whether the control condition was passive (i.e. waiting list or no intervention) or active/sham (control condition consisting of an activity of similar duration and intensity to the intervention condition but lacking the putatively effective component) [22, 23] (aspect of performance bias); (b) whether the outcome variable was measured by subjective rating (e.g. by questionnaires, which are regarded as susceptible to influences of reporting biases) or by a neuropsychological test (which is regarded as less susceptible to introduction of bias) [23] (aspect of detection bias), (c) whether a blinded (assessor not aware of participants’ treatment condition) or unblinded assessment of the outcome variable was conducted [22, 23] (aspect of detection bias), and (d) whether there was a risk of attrition bias. To assess the influences of these design conditions on the effect sizes, we distinguished between studies showing “high internal validity” (i.e. with an active control condition, and measurement of the outcome variables by neuropsychological tasks or subjective ratings by an assessor who was blind to the allocation of the child to intervention vs. control condition) and studies in which not all of these criteria were fulfilled. For control purposes, we calculated the weighted mean effect sizes for the highly valid studies separately and tested the moderator effect of the “internal validity” variable.

**Table 2:** Design characteristics and risk of bias of included studies

Study	Randomization or cluster-randomization^1^	Active control condition^1^	Assessment of outcomes^2^	Blinding of outcome assessments^1^	Unacceptable reasons for missing data^3^
Diamond et al. [30]	X	X	ICc: L	0	L
Ford et al. [31]	X	0	WM: L; ICc: L	X	UC
Nutley et al. [32]	X	X	WM: L	X	L
Tominey and McClelland [33]	X	0	ICc: L	X	L
Röthlisberger et al. [34]a	X	0	WM: L; ICc: L; FL: L	0	UC
Röthlisberger et al. [34]b	X	0	WM: L; ICc: L; FL: L	0	UC
Blair and Raver [18]	X	0	WM: L; ICc: L; FL: L	0	L
van Dongen et al. [35]	X	X	WM: L	X	L
Kroesbergen et al. [36]	X	0	WM: L	0	UC
Pears et al. [37]	X	0	ICc: L	0	UC
Blakey and Carroll [38]	X	X	WM: L; ICc: L	0	UC
Dias et al. [39]	X	0	ICc: L; FL: L; ODD: H; ADHD: H	0	UC
Flook et al. [40]	X	0	ICc: L; ICh: L; FL: L	0	UC
Liu et al. [41]	X	X	ICc: L	0	UC
Re et al. [42]a	X	0	WM: L; ICc: L	0	UC
Re et al. [42]b	X	0	WM: L; ICc: L	0	UC
Schmitt et al. [43]	X	0	ICc: L; FL: L	X	L
Tamm and Nakonezny [44]	X	0	WM: H; ICc: H; FL: H; ADHD: H	0	L
Traverso et al. [45]	X	0	WM: L; ICc: L; ICh: L; FL: L	X	L
Volckaert and Noël [46]	X	X	WM: L; ICc: L; ADHD: H; ODD: H	0	UC
Fishbein et al. [47]	X	0	ICc: L; ICh: L; ADHD: H; ODD: H	0	UC
Graziano and Hart [48]	X	0	WM: L; ICc: L; ODD: H	0	L
Murray et al. [49]	X	0	ICc: L; ICh: L	0	UC
Poehlmann et al. [50]	X	0	ICc: L	X	UC
Thibodeau et al. [51]	X	X	WM: L; ICc: L; FL: L	X	UC
Dias and Seabra [52]	X	X	FL: L; ADHD: H; ODD: H	0	L
Gade et al. [53]a	X	X	WM: L	0	UC
Gade et al. [53]b	X	X	WM: L	0	UC
Gade et al. [53]c	X	X	WM: L	0	UC
Gade et al. [53]d	X	X	WM: L	0	UC
Houssa et al. [54]	X	0	WM: H; ICc: H; ODD: H	0	UC
Howard et al. [55]a	X	X	WM: L; ICc: L; FL: L	0	L
Howard et al. [55]b	X	X	WM: L; ICc: L; FL: L	0	L
Joekar et al. [56]	X	0	ADHD: H	0	L
Upshur et al. [57]	X	0	WM: L; ICc: L	X	UC

^1^X = yes, 0 = no

^2^*L* low: use of neuropsychological tests, *H* high: use of ratings by unblinded parents or teachers; according to [25]

^3^*L* low risk of bias, *UC* unclear

For the assessment of publication bias, we used funnel plots and Egger’s test for funnel plot asymmetry. As funnel plot asymmetry can be caused by heterogeneity of effect sizes [25], we controlled for significant moderator variables in cases of significant heterogeneity (i.e. generated the funnel plots on the mixed model analyses) [26].

### Statistical analysis

#### Summary measures

The difference between the standardized mean change for the treatment and control group (*d* coefficient) was used as the primary effect size metric. According to Morris [27] and in line with the above-mentioned meta-analyses by Cortese et al. [23] and Sonuga-Barke et al. [22], the pooled pre-test standard deviations were used as estimates of the population variance. In two cases, however, the difference between groups in the post-score, was transformed into the d coefficient.

#### Synthesis of results

To assess the overall effects of the interventions on the EF domains (i.e. WM, cool IC, hot IC, FL) and on ADHD and ODD/externalizing symptoms, random effects models were used because between-studies variability of effect sizes is assumed. The amount of heterogeneity was estimated with the restricted maximum-likelihood estimator and tested by Cochran’s *Q* test [26].

#### Additional pre-specified analyses

To explore differences between the four approaches, we used mixed effects analyses. For the analyses of other moderator effects, mixed effects analyses also were performed. Calculations were carried out with “metafor” version 1.9–2 of R version 3.1.0 [26].

#### Overall procedure

The procedure was as follows: hypothesis 1: (1) for each of the EF outcome domains (WM, cool IC, hot IC, FL), the overall weighted mean effect size of the interventions was estimated. Heterogeneity of effect sizes was tested. (2) The weighted mean effect size for the studies with ADHD/ODD samples was estimated and compared with the mean effect in the general population samples. (3) The weighted mean effect size for the internally valid studies was estimated. (4) Differences between intervention approaches were explored. (5) Further moderator effects were tested if heterogeneity of the overall weighted mean effect size was significant. Hypothesis 2: (1) for the two outcome domains (ADHD symptoms, ODD/externalizing symptoms), the overall weighted mean effect sizes in the studies with ADHD/ODD samples were estimated. Heterogeneity of effect sizes was tested. (2) The weighted mean effect size for the internally valid studies was estimated. (3) Differences between intervention approaches were explored. (4) The moderator effects were tested if heterogeneity was significant.

## Results

**Fig. 1:**
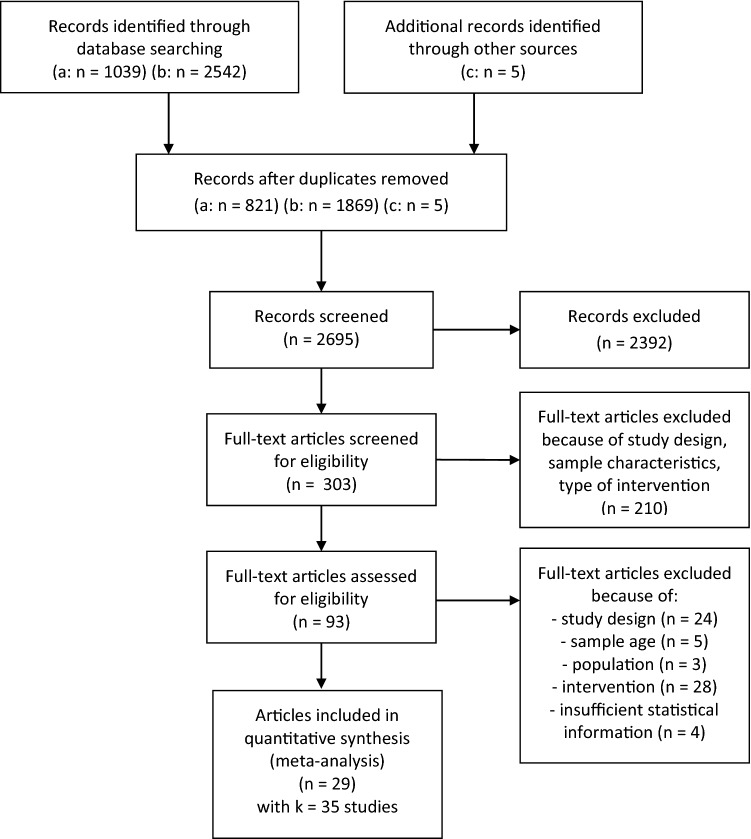
Prisma flowchart. **a** Search for keywords for intervention combined with keywords for executive functions, **b** search for keywords for intervention combined with key words for ADHD/ODD/externalizing symptoms, **c** other sources

**Fig. 2:**
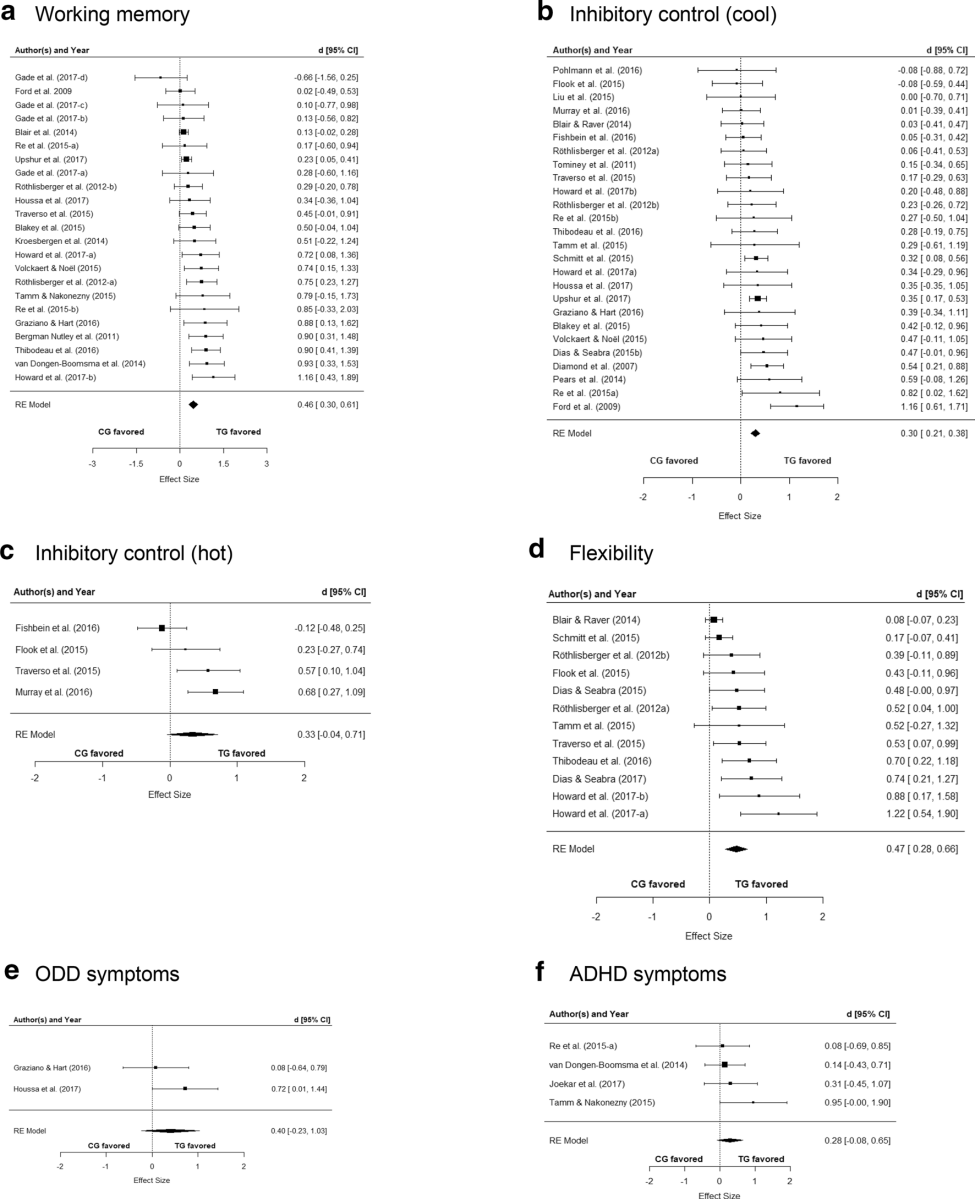
Forrest plots with *d* coefficients [27] for the meta-analyses of the four EF, the ADHD, and the ODD/externalizing outcome domains; *CG* control group, *TG* treatment group, *RE* random effect

Figure 1 shows the search processes. After removal of duplicates in the two parts of the search, 2695 records remained and were screened based on the abstract or the full text. In cases of insufficient statistical information, the information was requested from the authors. The procedure resulted in the inclusion of *k* = 35 independent RCTs (*k* = 35) reported in 29 articles. In all, *n* = 3068 children participated in these studies. Table 1 provides information on study design and sample characteristics, assessment of outcome variables and type of intervention for all included studies. Six of the studies analyzed samples of children with ADHD, ODD, or externalizing symptoms. Outcome variables included WM in 23 studies, cool IC in 26 studies, hot IC in four studies, and FL in 12 studies. Of the six studies on children with ADHD, ODD, or externalizing symptoms, four tested the effectiveness of an EF intervention for symptoms of ADHD, and two studies tested the effectiveness of an EF intervention for ODD/externalizing symptoms (Fig. 2).

### Working memory

In 23 studies, the effect of a cognitive training intervention on WM was analyzed. Fourteen studies focused on direct WM training, seven on cognitive scaffolding, no study on attention-directing strategies, and two studies analyzed programs with a minor EF component (Table 3). Eleven studies used an active control condition and assessed the outcome using neuropsychological tests.

**Table 3:** Weighted mean effects of cognitive training on EFs

		Working memory	Inhibition (cool)	Inhibition (hot)	Flexibility
All studies	*d*; 95% CI	0.46***; 0.30–0.61	0.30***; 0.21–0.38	0.33*; − 0.04 to 0.71	0.47***; 0.28–0.66
Number of studies	*k*	23	26	4	12
Heterogeneity	*I*^2^ *Q*(*df*)	49.4% 42.5(22); *p* = 0.005	0% 25.9(25); ns	66.5% 9.6(3); *p* = 0.02	56.9% 27.2(11), *p* = 0.004
Internally valid studies	*d*; 95% CI	0.60***; 0.35–0.85	0.39***; 0.19–0.58	–	0.84***; 0.55–1.12
Number of studies	*k*	11	7	0	4
Heterogeneity	*I*^2^ *Q*(*df*)	36% 17.2(10); ns	0% 2.57(6); ns	–	0% 1.67(3); ns
ADHD/ODD samples	Moderator effect	*Q*(1) = 0.94, ns	*Q*(1) = 0.69, ns	–	–
Yes	*d*; 95% CI; *k*	0.64***; 0.31–0.96; 5	0.46*; 0.07–0.84; 4	–	0.52; − 0.27 to 1.32; 1
No	*d*; 95% CI; *k*	0.42***; 0.25–0.60; 18	0.29***;0.19–0.38; 22		0.47***;0.27–0.67; 11
Intervention approaches	Moderator effect:	*Q*(1) = 0.43; ns	*Q*(2) = 3.99^a^, ns	*Q*(2) = 6.48^b^, *p* = .039	*Q*(1) = 0.14^a^; ns
Direct	*d*; 95% CI; *k*	0.52***; 0.31–0.72; *k* = 14	0.27***; 0.12–0.42; *k* = 10	– *k* = 0	0.55**; 0.19–0.90; *k* = 5
Cognitive scaffolding	*d*; 95% CI; *k*	0.42**; 0.14–0.69; *k* = 7	0.42***; 0.20–0.63; *k* = 9	0.57*; 0.10–1.04; *k* = 1	0.40**; 0.18–0.72; *k* = 6
Attention direction	*d*; 95% CI; *k*	–, –; *k* = 0	0.01; − 0.39 to 0.41; *k* = 1	0.68**; 0.27–1.09; *k* = 1	0.43; − 0.11 to 0.96; *k* = 1
Program with EF component	*d*; 95% CI; *k*	0.45; − 0.15 to 1.04; *k* = 2	0.23*; 0.04–0.43; *k* = 6	0.02; − 0.32 to 0.35; *k* = 2	– –; *k* = 0

*ns* not statistically significant, *95% CI* 95% confidence interval, *k* number of studies

^a^Intervention approaches with *k* = 1 not considered

^b^Comparison between the two programs with EF component and the two other interventions

The overall mean effect size of the 23 studies proved to be statistically significant and was of almost medium strength (*d* = 0.46; *p* < 0.001). Inter-study heterogeneity of effect sizes was significant (Table 3). In *k* = 5 studies with samples of children showing high symptoms or a diagnosis of ADHD or ODD/externalizing disorders, the mean effect size was *d* = 0.64 (*p* < 0.001). The mean effect size of these studies did not differ from that in studies of children from the general population (Table 3). In the *k* = 11 studies categorized as “highly valid”, the mean effect size was *d* = 0.60 (*p* < 0.001). The moderator effects by internal validity was statistically significant (*Q*(1) = 4.94; *p* = 0.026; *k* = 23), indicating a higher mean effect size of the more rigorously controlled studies. Intervention approaches did not significantly differ (Table 3).

**Fig. 3:**
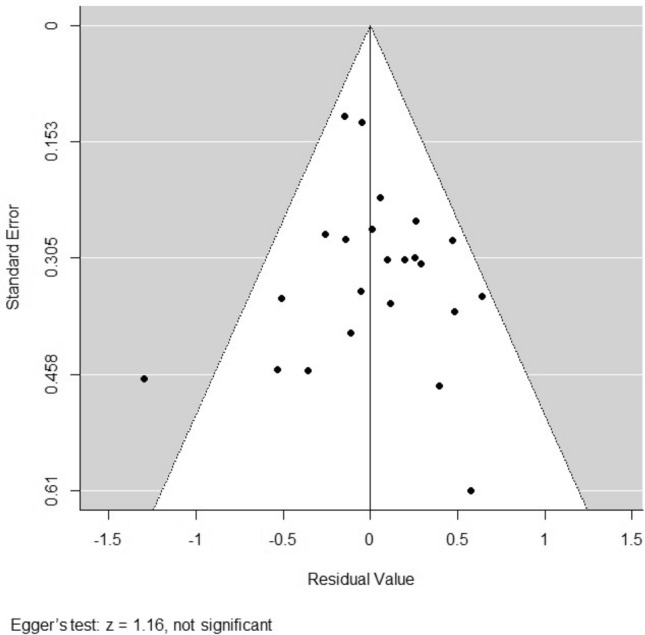
Funnel plot for the mixed model meta-analysis on working memory (controlling for the moderator effect by study validity)

Due to the significant overall heterogeneity of the effect sizes, potential moderator effects by sample and intervention characteristics were analyzed. We found no significant moderator effects by percentage of boys (*Q*(1) = 0.04, *k* = 22) or mean age of sample (*Q*(1) = 0.21, *k* = 23). Among the characteristics of the intervention, there were also no significant moderator effects (delivery by trainer/therapist vs. teacher: *Q*(1) = 2.17, *k* = 23, duration of intervention in weeks: *Q*(1) = 2.71, *k* = 23, intensity: *Q*(1) = 0.42, *k* = 21, group vs. single-child setting: *Q*(1) = 0.00, *k* = 23). The funnel plot for the mixed model meta-analysis (controlling for the moderator effect by study validity) did not show significant asymmetry (Fig. 3, Appendix).

### Inhibitory control

In 26 studies, the effectiveness of cognitive training interventions on non-reward-related, cool IC was examined (10 focusing on direct IC training, nine on cognitive scaffolding, one on attention-directing strategies, and six analyzed programs with minor EF components). Seven of the 26 studies were classified as highly valid.

The overall effect was *d* = 0.30 (*p* < 0.001), with no significant inter-study heterogeneity of effect sizes (Table 3). In the studies with ADHD/ODD samples, the mean effect size was *d* = 0.46 (*p* = 0.015). In the highly valid studies, the mean weighted effect size was *d* = 0.39 (*p* < 0.001). Moderator effects by study sample (*Q*(1) = 0.69) and validity (*Q*(1) = 0.72) were not significant. Effect sizes of the three intervention approach categories (with more than one study) did not significantly differ (Table 3).

**Fig. 4:**
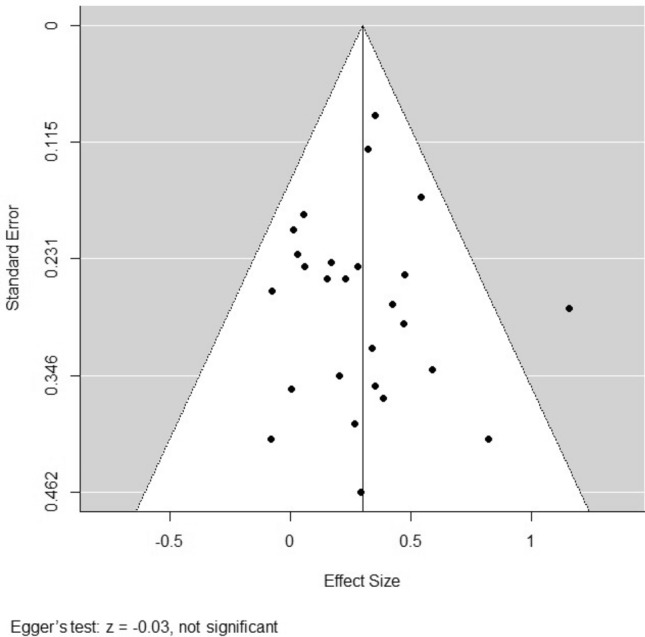
Funnel plot for the meta-analysis on cool inhibitory control

Given the non-significant heterogeneity, we refrained from further moderator analyses. There was no indication of funnel plot asymmetry (Fig. 4, Appendix).

### Reward-related inhibitory control

Four studies examining the effect of cognitive training on reward-related, hot IC were included, one focusing on cognitive scaffolding, one analyzed attention-directing strategies and two programs had a minor EF component. There was no study with ADHD/ODD samples. No study fulfilled all validity criteria. However, all studies used neuropsychological tests to assess the hot IC outcome. The weighted mean effect size of the four studies approached statistical significance (*d* = 0.33, *p* < 0.10). Between-study heterogeneity of effect sizes was significant (Table 3). We compared the interventions with a minor EF component with the two other intervention approaches. The difference was statistically significant indicating stronger improvement of hot IC by attention-directing and cognitive scaffolding strategies (Table 3).

Further moderator analyses yielded no significant results (mean age of sample: *Q*(1) = 0.21; percentage of boys: *Q*(1) = 0.76; duration of intervention: *Q*(1) = 0.85; doses/intensity: *Q*(1) = 0.01; delivery (teacher vs. trainer/therapist): *Q*(1) = 0.09; all interventions used a group setting). An analysis of funnel plot asymmetry was not appropriate due to the low number of studies.

### Flexibility

Twelve studies which tested the effect of a cognitive intervention on FL were identified; five on direct EF training including FL, six on programs focusing on scaffolding, and one on attention-directing interventions. Four studies were categorized as highly valid.

The overall weighted mean effect size was *d* = 0.47 (*p* = 0.001). Heterogeneity was statistically significant (Table 3). There was only one study on children with ADHD symptoms (Table 3). The four well-controlled studies yielded a large, statistically significant mean effect size of *d* = 0.84 (*p* < 0.001). The moderator effect by validity of the study design was significant (*Q*(1) = 9.40, *p* < 0.01), indicating larger effects of the more rigorously controlled studies. No significant differences emerged between the intervention approaches (see Table 3).

**Fig. 5:**
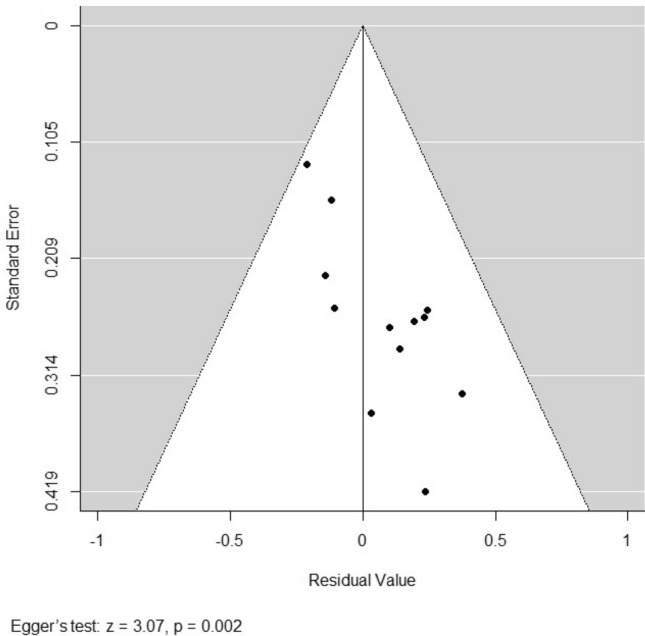
Funnel plot for the mixed model meta-analysis on flexibility (controlling for the moderator effect by study validity)

Further moderator analyses yielded no statistically significant effects (mean age of sample: *Q*(1) = 1.17; percentage of boys: *Q*(1) = 0.59; duration of intervention *Q*(1) = 2.02; intensity of intervention: *Q*(1) = 0.03; setting: *Q*(1) = 0.26; delivery: *Q*(1) = 0.15). When controlling for the significant moderator effect by study validity, the Egger test for funnel plot asymmetry was significant (Fig. 5, Appendix), indicating that an influence of publication bias on the effects on flexibility is probable.

### ODD/externalizing symptoms

In two studies, the effects on ODD/externalizing symptoms in samples of children with ADHD, ODD, or externalizing symptoms were analyzed (Table 4). In both trials, preschool children were enrolled if they scored above a clinical cut-off on an externalizing behavior problem questionnaire scale. One study focused on cognitive scaffolding and one analyzed a program with a minor EF component (Table 1). No study used an active control condition or a blinded assessment of the ODD/externalizing symptoms outcome. The weighted mean effect size of the two studies was *d* = 0.40 but did not reach statistical significance. Due to the low number of studies, it was not possible to statistically compare the intervention approaches. Notably, however, the RCT which analyzed scaffolding techniques showed a significant, large mean effect size of *d* = 0.72 (*p* < 0.05). The other RCT analyzed a program with a minor EF component. The effect was not significant (*d* = 0.08, Table 4). Analysis of funnel plot asymmetry was inappropriate due to the low number of studies.

**Table 4:** Effects of cognitive training on ODD and ADHD symptoms in children with diagnoses or high symptoms of ADHD or ODD/externalizing disorders

Weighted mean effect sizes		ODD symptoms	ADHD symptoms
All studies	*d*; 95% CI	0.40; − 0.23 to 1.03	0.28; − 0.08 to 0.64
Number of studies	*k*	2	4
Heterogeneity	*I*^2^ *Q*(*df*)	– –	0% 2.41(3); ns
Internally valid studies	*d*; 95% CI	–	0.45; − 0.32 to 1.23
Number of studies	*k*	0	2
Heterogeneity	*I*^2^ *Q*(*df*)	– –	51.07% 2.04(1); ns
Analyses of moderator effects			
Intervention approaches	*Q*(*df*)	–	–
Direct	*d*; CI_95_	– –; *k* = 0	0.17; − 0.22 to 0.56; *k* = 3
Cognitive scaffolding	*d*; CI_95_	0.72*; 0.01–1.44; *k* = 1	0.95*; 0.00–1.90; *k* = 1
Attention direction	*d*; CI_95_	– –; *k* = 0	– –; *k* = 0
Program with EF component	*d*; CI_95_	0.08; − 0.64 to 0.79; *k* = 1	– –; *k* = 0

*ns* not statistically significant; *p < 0.05; **p < 0.01; ***p < 0.001

### ADHD symptoms

In four studies, the effect of a cognitive training intervention (three focusing on direct EF training, one on cognitive scaffolding) on the ADHD symptoms of preschoolers with high symptoms or a diagnosis of ADHD, ODD, or externalizing disorders was analyzed (Table 4). Two studies included children with ADHD diagnoses, one studies children with high symptoms of ADHD, and one study children with high symptoms of externalizing behavior problems. The weighted mean effect size of these studies was *d* = 0.28 (*p* = 0.13). There was no significant heterogeneity of the effect sizes of the studies. No study fulfilled all validity criteria. However, one study used a blinded assessment of the ADHD outcome and another study used an active control condition (Table 1). In these two studies, the weighted mean effect was *d* = 0.45, but did not reach statistical significance due to the large differences between the effect sizes and the relatively small sample sizes. As there was only one study which used cognitive scaffolding, the difference between intervention approaches was not tested (Table 4). Notably, the RCT on a cognitive scaffolding intervention yielded a large, significant mean effect of *d* = 0.95; *p* < 0.05) while the mean effect size of the three RCTs on direct EF training was *d* = 0.17 (not significant). Analysis of funnel plot asymmetry was inappropriate due to the low number of studies.

## Discussion

EFs have been found to predict social adjustment development in childhood and adolescence and are assumed to be causally involved in the pathogenesis of ADHD and ODD. The core EFs of WM, IC, and FL show major normative developmental improvements in preschool years. They are presumed to be particularly malleable by cognitive training interventions in this developmental period. The number of studies analyzing this hypothesis has increased considerably in recent years. However, this research has not yet been systematically reviewed and summarized by means of a meta-analysis.

We analyzed the effectiveness of EF training interventions developed specifically for preschool children regarding the improvement of core EFs and the reduction of ADHD and ODD symptoms. The main findings were as follows: based on the RCTs with the lowest risk of bias (i.e. categorized as highly internal valid), we found a significant, medium effect on WM, and a significant small to medium effect on cool IC. There were no highly valid studies on hot IC and only four on FL. In preschoolers with ADHD/ODD (symptoms), significant (almost) medium-sized effects on WM and cool IC emerged. With the exception of hot IC, there were no differences between the intervention approaches. Hot IC, however, showed greater improvements from interventions focusing on attention-directing strategies and cognitive scaffolding. In children with ADHD/ODD (symptoms), overall small to medium sized, not statistically significant effects on ODD and ADHD symptoms emerged. Effects of cognitive scaffolding programs were large, while the effects of programs using direct training of EFs or programs with a minor EF component were negligible.

### Executive functions

The overall weighted mean effect sizes in the four EF domains ranged between *d* = 0.30 (cool IC) and *d* = 0.47 (FL). The effect on WM (*d* = 0.46, *p* < 0.001) is comparable with the findings of meta-analyses on WM/attention training by Cortese et al. [23] (verbal WM: *d* = 0.51, *p* < 0.05; visual WM: *d* = 0.47, *p* < 0.05) in children/adolescents with ADHD and by Melby-Lervåg and Hulme [19] (visuospatial WM: *d* = 0.46, *p* < 0.01) in children from diverse populations. In the present meta-analysis, in all instances, the mean effect size was larger in the trials with the highest internal validity (low risk of bias). In the domains of WM and FL, the moderator effect by study validity reached statistical significance. This finding is unsurprising given that controlling for unsystematic errors leads to a closer link between the experimental conditions and the outcome variable. Moreover, the finding underscores the methodological quality (i.e. no systematic biases in favor of the interventions) of the included studies. This latter aspect is probably due to the use of neuropsychological tests (i.e. no subjective ratings) for the assessment of the outcomes. Overall, it can be concluded that the interventions lead to a substantial improvement in preschoolers’ WM and cool IC. Due to publication bias, however, the effect on FL should be interpreted with caution.

#### Intervention approaches

We found no significant differences between the intervention approaches in the cool EF domains, i.e. approaches resulted in comparable effects on WM, cool IC, and FL. The number of studies in the hot IC domain was rather limited, which is likely due to the more recent recognition of hot IC as a distinct component of IC (see e.g. [11]). Nevertheless, in this domain, the exploratory analyses revealed a significant difference between intervention approaches, indicating better results from programs focusing on attention-directing strategies and cognitive scaffolding compared to social skills/emotion regulation programs with a minor EF component. Hot IC involves the top-down control of motivational impulses, e.g. to approach a rewarding stimulus. As has been shown by experiments using the delay-of-gratification paradigm, strategic attention deployment is most effective when waiting for a reward and resisting the temptation to approach is demanded. The strategy usually emerges spontaneously in later preschool years but can also be conveyed by verbal instructions to the child (see [28, 29]). Thus, it might be that preschoolers learn to effectively use attention-directing strategies in waiting situations relatively easily. However, more research on this topic is needed before any secure conclusions can be drawn.

#### ODD and ADHD symptoms

In previous research, transfer effects of improvements in EFs after direct (often computerized) cognitive training were rather low. Even if participants increased, for instance, their WM capacity, generalization to other EFs and cognitive abilities (near transfer effects) or to academic skills and subjective ratings of e.g. ADHD symptoms (far transfer effects) were poor [20, 22]. Many of the preschool interventions, however, focus on multiple core EFs. Therefore, it is difficult to isolate near transfer effects to other EFs. The far transfer effects of the programs regarding ADHD, ODD/externalizing symptoms which were addressed in the present analysis are discussed below.

In the preschool period, during which ADHD and ODD symptoms emerge, the not yet full-blown disorders are hard to distinguish. ADHD and ODD symptoms often occur together and only over the course of time do they differentiate into the subtypes of ADHD, ODD, and the combination thereof [16]. For this reason, we merged research on preschoolers who showed high symptoms and those who already had diagnoses of ADHD and ODD, but analyzed the effects of the interventions on ADHD and ODD/externalizing symptoms separately due to the possibility of differing effects in the domains. In all, six studies involved children with ADHD, ODD, or externalizing symptoms. As mentioned, in these studies, the effects on WM and cool IC were statistically significant and of medium strengths.

In samples of children with ADHD and ODD/externalizing symptoms, the overall effects on the ADHD symptoms (four RCTs) and the ODD/externalizing symptoms (two RCTs) were not statistically significant. In both domains, a RCT on a cognitive scaffolding program showed significant, large effects, while effects of direct (computerized) training of EFs or a program with a minor EF component were negligible and not statistically significant. Cognitive scaffolding programs usually aim at multiple EFs, comprise elements which target the facilitation of transfer to everyday life, and take place in everyday interpersonal contexts. These programs might, therefore, be better tailored to the probably multiple EF deficits of children with ADHD and ODD, and might better represent the everyday context in which the symptoms appear.

Nevertheless, these conclusions need to be seen as tentative. Of the six RCTs, only two RCTs on ADHD samples showed good internal validity (one on direct WM training, one on a cognitive scaffolding program). Even though these two well-controlled studies showed an almost medium effect size on average, statistical significance was not reached due to large differences in the single effects. More research is needed in this area. Based on our results, RCTs on cognitive scaffolding programs with blinded assessments of the ADHD and ODD outcomes would be particularly worthwhile.

## Limitations and conclusions

As mentioned above, some findings of the meta-analysis are limited by the insufficient number of eligible studies. Specifically, more studies are needed which use blinded assessments of subjective ratings of ADHD and ODD symptoms, and analyze the effects on hot IC. Moreover, it is necessary to analyze the mediation effects by the core EFs on the ADHD and ODD/externalizing outcomes on the level of single studies. As the number of studies on this topic is insufficient, the question of whether the improved EFs actually lead to a reduction of the ExtD symptoms must remain unanswered. The results of the current meta-analysis are compatible with such mediation processes (as effect sizes on EFs and ADHD symptoms were significant) but, as mentioned, they do not prove mediation. Regarding FL, we found indications of publication bias. As shown by the funnel plot (Appendix), there were relatively few small studies reporting small, non-significant effects. The finding points to the need for the publication of well-controlled trials with FL outcome measures, irrespective of the significance of the results.

To conclude, although we found many well-controlled RCTs on the effectiveness of preschool interventions aiming at EFs, more research is needed. The meta-analysis revealed significant, mostly medium-sized effects of the preschool interventions on core EFs in studies showing a low risk of bias. In children with ADHD and ODD, cognitive scaffolding interventions were most effective in terms of reducing ADHD and ODD symptoms. However, these findings need to be confirmed in future research. Such research appears to be worthwhile given the indications that these interventions could adequately support preschool children who show ADHD/ODD (symptoms).

## Appendix

See Figs. 3, 4 and 5.
